# Smart triage: Development of a rapid pediatric triage algorithm for use in low-and-middle income countries

**DOI:** 10.3389/fped.2022.976870

**Published:** 2022-11-22

**Authors:** Alishah Mawji, Edmond Li, Dustin Dunsmuir, Clare Komugisha, Stefanie K. Novakowski, Matthew O. Wiens, Tagoola Abner Vesuvius, Niranjan Kissoon, J. Mark Ansermino

**Affiliations:** ^1^ Department of Anesthesiology, Pharmacology & Therapeutics, University of British Columbia, Vancouver, BC, Canada; ^2^ Centre for International Child Health, BC Children’s Hospital Research Institute, Vancouver, BC, Canada; ^3^ WALIMU, Kampala, Uganda; ^4^ Department of Pediatrics, Jinja Regional Referral Hospital, Jinja, Uganda; ^5^ Department of Pediatrics, University of British Columbia, Vancouver, BC, Canada

**Keywords:** sepsis, triage, low-and-middle income countries, prediction, model, children, logistic regression

## Abstract

**Introduction:**

Early and accurate recognition of children at risk of progressing to critical illness could contribute to improved patient outcomes and resource allocation. In resource limited settings digital triage tools can support decision making and improve healthcare delivery. We developed a model for rapid identification of critically ill children at triage.

**Methods:**

This was a prospective cohort study of acutely ill children presenting at Jinja Regional Referral Hospital in Eastern Uganda. Variables collected in the emergency department informed the development of a logistic model based on hospital admission using bootstrap stepwise regression. Low and high-risk thresholds for 90% minimum sensitivity and specificity, respectively generated three risk level categories. Performance was assessed using receiver operating characteristic curve analysis on a held-out test set generated by an 80:20 split with 10-fold cross validation. A risk stratification table informed clinical interpretation.

**Results:**

The model derivation cohort included 1,612 participants, with an admission rate of approximately 23%. The majority of admitted patients were under five years old and presenting with sepsis, malaria, or pneumonia. A 9-predictor triage model was derived: logit (*p*) = −32.888 + (0.252, square root of age) + (0.016, heart rate) + (0.819, temperature) + (−0.022, mid-upper arm circumference) + (0.048 transformed oxygen saturation) + (1.793, parent concern) + (1.012, difficulty breathing) + (1.814, oedema) + (1.506, pallor). The model afforded good discrimination, calibration, and risk stratification at the selected thresholds of 8% and 40%.

**Conclusion:**

In a low income, pediatric population, we developed a nine variable triage model with high sensitivity and specificity to predict who should be admitted. The triage model can be integrated into any digital platform and used with minimal training to guide rapid identification of critically ill children at first contact. External validation and clinical implementation are in progress.

## Introduction

Child mortality in low- and-middle income countries (LMICs) remains high (1). Most of these deaths are preventable or easy to treat and occur within 24 h of hospital admission (2). Rapid identification of critical illness is important because delay in treatment is associated with an escalated risk of death and disability (2). Triage, the prioritization of patients to identify the sickest for earliest intervention, can optimize resource allocation and markedly improve patient outcomes especially in resource limited settings (3–5).

The validity of current pediatric triage tools is uncertain (6, 7), particularly within resource limited settings. A lack of standardized data and insufficient follow-up on children not admitted to health facilities remain major limitations of research in this field. The current gold standards for triage are the World Health Organization's (WHO) Integrated Management of Childhood Illness (IMCI) (8) and Emergency Triage Assessment and Treatment Plus (ETAT+) (9) guidelines for diagnosing and managing sick patients. However, health workers in resource limited settings often lack the training and infrastructure to follow these clinical recommendations (10, 11).

Digital health interventions have the potential to support clinical decision making and improve the quality of care in resource limited settings (12). Digital platforms can facilitate quality improvement, and data-driven algorithms can be continuously updated and improved with new information or optimized to meet the specific needs of each setting (13). It has been demonstrated that digital triage tools can optimize resource allocations, reduce treatment wait times, and improve patient outcomes (14).

We describe the development of a simple triage model combining clinical signs, symptoms, and readily available vital signs that can be embedded into a digital platform. The model was designed for use with limited clinical training to guide rapid identification of critically ill children at the first point of contact. Model development was the first phase in the Smart Triage study. The model has since been implemented in a mobile triage platform and is being used at hospital sites in Kenya and Uganda with the goal of reducing time to treatment in children.

## Methods

Model development and reporting followed TRIPOD (transparent reporting of a multivariable prediction model for individual prognosis or diagnosis) guidelines (Supplementary File S1) (15).

### Trial registration

Clinical Trials.gov Identifier: NCT04304235, Registered 11 March 2020.

### Study design and setting

This study was conducted at the pediatric emergency department (ED) of Jinja Regional Referral Hospital (JRRH), a public hospital funded by the Uganda Ministry of Health. JRRH is the largest referral hospital in Eastern Uganda and serves patients residing in Jinja and eight surrounding districts**.** In 2021, the pediatric ED evaluated 29,022 patients and had an admission rate of approximately 20%. There are 87 admission beds in the unit. Most children who present to the pediatric ED are under five years of age.

Triage is conducted by one designated nurse with assistance from two to three student nurses.

This was a prospective cohort study conducted between April 2020 and March 2021. This study was approved by the institutional review boards at the University of British Columbia in Canada (ID: H19-02398; H20-00484), the Makerere University School of Public Health in Uganda and the Uganda National Council for Science and Technology. This is one modelling component of a multi-site clinical study for which the full protocol has been published (16).

### Eligibility and sampling

Children under 19 years of age seeking assessment for an acute illness at the pediatric emergency department of JRRH between 8:00 am and 5:00 pm were enrolled. Children presenting for elective procedures, scheduled appointments or treatment of chronic illnesses were not eligible for enrollment. Participation was voluntary and written informed consent was provided by a parent or guardian prior to enrollment. Assent was required from children above eight years of age. A quasi-random sampling method based on time cut-offs was adopted (17).

### Data collection and management

Study procedures were initiated in the triage waiting area of the pediatric OPD. Following enrollment, study nurses conducted an interview and clinical examination to capture the candidate predictor variables (Supplementary Table S2). Study procedures did not interfere with or introduce delays to care delivery. Data were collected using password secured Android tablets, and a custom-built mobile application with an encrypted database. The Masimo iSpO2® Pulse Oximeter with micro USB connector was connected directly to the tablet to collect pulse oximetry and heart rate and the SureTemp 692 thermometer was used to measure core temperature. At the end of each day, data was uploaded directly from the Android tablets to REDCap (Research Electronic Data Capture) (18) and sent to the central study server at the BC Children's Hospital Research Institute. After each upload, data on the tablets was automatically deleted. Standard operating protocols for data collection and management are available on the Pediatric Sepsis CoLab Dataverse (17).

### Candidate predictor variables

Predictor variables were based on sepsis, the most common cause of preventable child deaths from infection (19). Candidate predictor variables were generated in accordance with the guidelines developed by the Pediatric Sepsis Predictor Standardization (PS2) working group (20, 21). The PS2 working group is a subgroup within the Pediatric Sepsis CoLab, a global network for collaboration and data sharing to improve the quality of care and decrease morbidity and mortality from sepsis (22). Guidelines for the standardized collection of predictor variables in studies for pediatric sepsis were derived using a systematic review and a modified Delphi approach to standardize definitions and assign a tier level to each variable using a three-point tiering system (Tier 1: essential, Tier 2: Important, Tier 3: Exploratory) (21, 23). Due to limitations in time and resources at the point of triage in resource limited settings, all Tier 1 variables and relevant Tier 2 variables (as determined by study investigators) applicable to children of all ages were selected for inclusion, for a total of 63 candidate predictor variables (Supplementary Table S2).

### Modelling outcome

The primary outcome was hospital admission (as determined by the hospital clinicians) for more than 24 h. Currently, need for admission at JRRH is determined using ETAT + guidelines and clinician expertise. To capture any critically ill cases who may have been sent home on the day of enrollment, children readmitted within 48 h were also considered to have a positive admission outcome. The hospital registry was used to collect data on admission status. Caregivers were called 7 days post-discharge, or 7 days post-study enrollment (for non-admitted patients), to confirm admission status and determine readmission status. Therefore, readmissions to other healthcare centers were also counted in our primary outcome.

### Statistical analysis

#### Sample size

The sample size estimation was computed based on the number of predictors expected in the final model (*n*), the outcome event rate (*I*), and calculated as *N* = (*n* × 10)/*I* by employing the typical minimum standard of 10 events per effective variable. Preliminary analysis at the study site suggested an estimated admission rate of 20%, and thus to allow for a model with 10 predictors we require a minimum sample of 500 participants. Based on the uncertainty commonly present in these smaller sample sizes and clinical feasibility (large case load), we targeted a minimum sample of 1,500 participants (24).

#### Model development, validation, and calibration

As missing data was expected to be minimal (<5%), median and mode imputation strategies were used for continuous and categorical predictors, respectively. Predictors with less than 10 events per variable were not considered for inclusion in the model to reduce potential of overfitting. Continuous variables were assessed graphically for linear associations with the outcome and transformed where appropriate. A physiological transformation of oxygen saturation was used to linearize the relationship between oxygen severity and impairment of gas exchange. Transformation was based on an altitude adaptive virtual shunt model [70.103 × log_10_(101.687 − SpO_2_) − 55.833], which has been demonstrated to improve the fit of logistic regression models (25, 26).

Bootstrapped stepwise regression was used to select predictors to comprise the logistic regression model. A stepwise selection procedure, which repetitively added or dropped predictors to minimize Akaike information criterion (AIC), was repeated on 2,000 bootstrap replicates (27). A final consensus model was derived from the predictors present in at least 80% of the bootstrap sample models. To quantify the predictive accuracy of the consensus predictors, data was split into training (80%) and validation (20%) sets and a 10-fold cross validation procedure was applied to compute a pooled estimate of the area under the receiver operating characteristic (AUROC) curve, sensitivity, specificity, predictive values, and likelihood ratios. Standard errors of these characteristics were derived from 2,000 bootstrap replicates. Calibration was evaluated graphically (28). The final model was applied and evaluated using the methods above on the subpopulation of children under five years of age to assess robustness across age groups. The model was performance was also evaluated on only children under 5 years and alternatively using all admissions (regardless of length of stay) as positive outcome to assess robustness.

#### Risk stratification and clinical interpretation

Two risk thresholds were selected to divide participants into three triage categories (emergency, priority, and non-urgent). Low and high-risk thresholds were selected to correspond with a sensitivity and specificity of at least 90%, respectively. These markers were selected to develop a triage model highly sensitive in detecting high risk cases (avoiding false negatives) but specific in identifying emergency cases (avoiding false positives) to optimize resource allocation while seeing that critically ill children do not experience delays. A risk stratification table was used to evaluate model classification accuracy, defined as the ability of the model to separate participants into triage categories, such that cases with and without outcomes are more likely to be in the higher and lower risk strata, respectively.

### Patient and public involvement

Patients were not involved in the conceptualisation, design or conduct of this study. The results of the study will not be disseminated directly to participants.

## Results

### Participants

**Figure 1:**
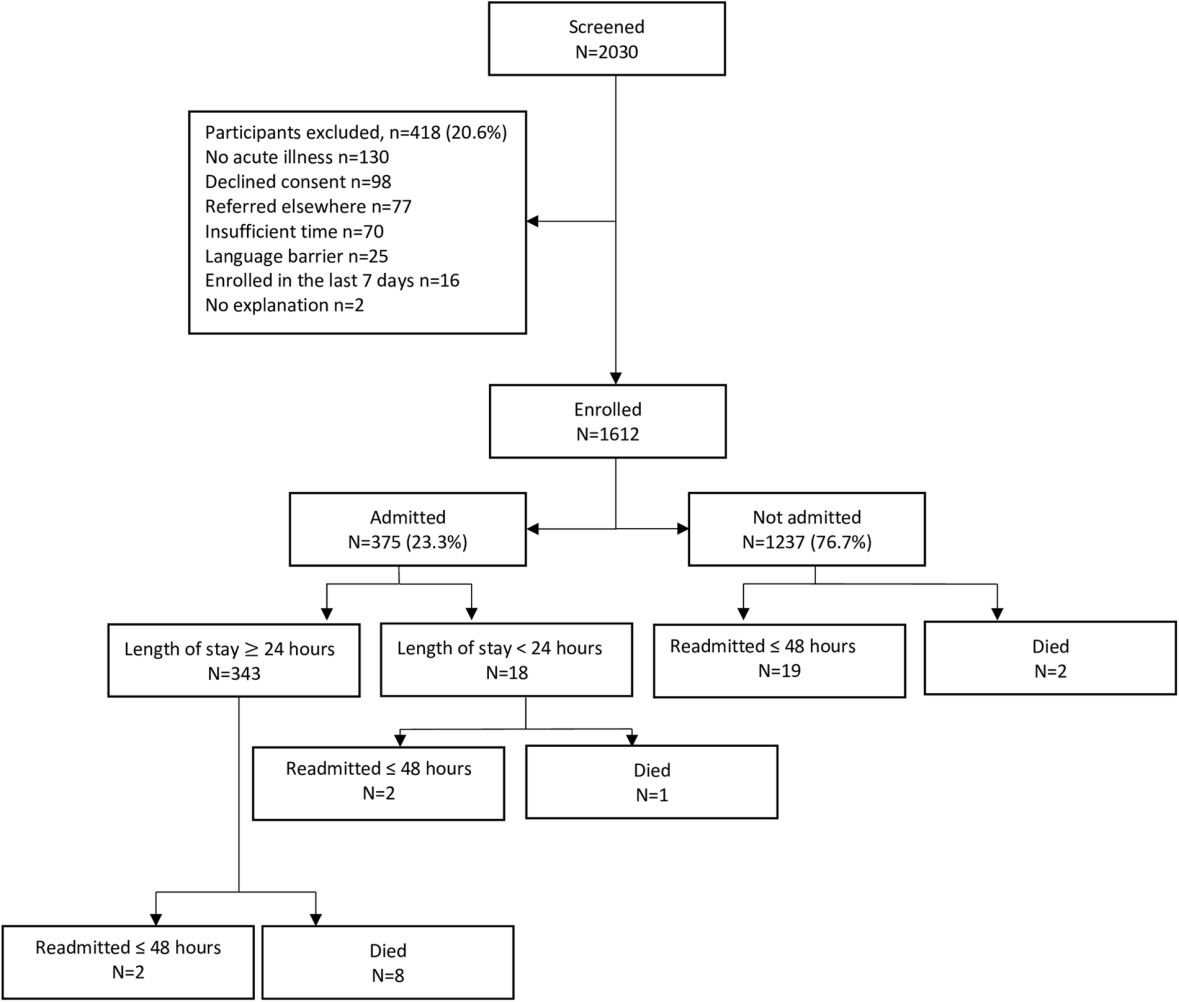
Diagram of participant flow.

During April 27th, 2020 and March 31st, 2021, 2,030 patients were screened for eligibility, of which 418 (20.6%) were excluded (Figure 1). In total, 1,612 participants were enrolled in the study. The prevalence of males and females was roughly equal and approximately 90% of the cohort was comprised of children under 5 years of age (Table 1). According to the anthropometric characteristics, 147 (12.3%) participants were underweight (weight-for-age *z*-score <−2), 325 (20.2%) were stunted (height-for-age *z*-score <−2), and 169 (10.5%) were wasted (weight-for-height *z*-score <−2) (Table 1).

**Table 1:** Participant characteristics.

	Participants, *N* (%)
**Sex**	
Female	780 (48.5)
Male	829 (51.5)
**Age**	
<30 days	80 (5.0)
30 days–1 year	633 (39.3)
1–2 years	406 (25.2)
2–5 years	318 (19.7)
5–10 years	144 (8.9)
10+ years	31 (1.9)
**Anthropometric Characteristics**	
MUAC < 125 mm	205 (12.7)
MUAC < 115 mm	87 (5.4)
Underweight (WAZ < −2)	198 (12.3)
Severely underweight (WAZ < −3)	75 (4.7)
Stunting (HAZ < −2)	325 (20.2)
Severe stunting (HAZ < −3)	147 (9.1)
Wasting (WHZ < −2)	169 (10.5)
Severe wasting (WHZ < −3)	81 (5.0)
Vital Signs	
Oxygen saturation < 90%	29 (1.8)
Respiratory rate > 60 breaths per minute	224 (13.9)
Temperature > 40°C	18 (1.1)
**Outcomes**	
Admitted	375 (23.3)
*Length of stay ≧24 h*	343 (21.3)
Readmitted	12 (0.7)
*Within 48 h*	4 (0.2)
Mortality	9 (0.6)
Not admitted	1,232 (76.4)
Returned and admitted	26 (1.6)
*Within 48 h*	19 (1.2)
Mortality	2 (0.1)
Positive admission outcome*	364 (22.6)
**Admission Diagnosis Profile**	
Malaria	170 (45.3)
Pneumonia	50 (13.3)
Sepsis	83 (22.1)
Neonatal Sepsis	10 (2.7)
Gastroenteritis/Diarrhea	18 (4.8)
Other	44 (11.7)

* Defined as admitted for at least 24 h or returned and admitted within 48 h. MUAC, middle upper arm circumference; WAZ, weight-for-age *z*-score; HAZ, height-for-age *z*-score; WHZ, weight-for-height *z*-score.

A total of 375 (23.3%) participants were admitted to the hospital, of which 343 (21.3%) were admitted for a minimum duration of 24 h and 4 (0.1%) were readmitted within 48 h of discharge (Table 1). Additionally, 19 (1.9%) participants initially sent home returned and were admitted to a hospital or health facility within 48 h. This resulted in 364 (22.6%) positive admission outcomes (Table 1). The most common reasons for admission were malaria (45.3% of admitted participants), sepsis (22.1%), and pneumonia (13.3%) (Table 1). Five variables did not meet the minimum criteria of 10 events for inclusion in the model derivation process (Supplementary Table S3). Univariate analysis revealed many variables to be significantly associated with the admission outcome and missing data was minimal (Supplementary Table S3).

### Triage model

**Figure 2:**
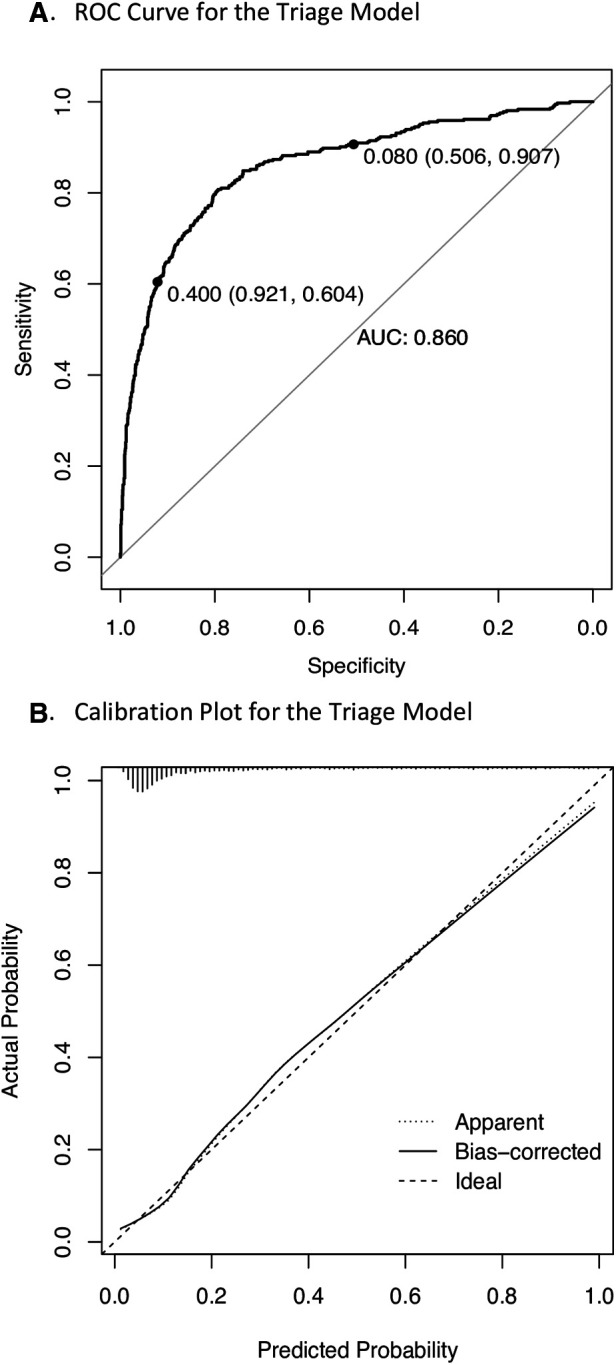
Performance of the triage model in the study cohort. ROC, receiver operating characteristics; AUC, area under the curve. (**A**) 10-fold cross validated ROC curve where labelled points represent low risk (8%) and high risk (40%) thresholds. (**B**) Calibration plot where 45-degree straight line corresponds to the line of perfect calibration on which model predicted risks coincide with the observed frequency and the bias corrected curve represents pooled results from 1,000 bootstrap samples.

The equation for the derived nine-predictor logistic triage model was: logit (*p*) = −32.888 + (0.252, square root of age) + (0.016, heart rate) + (0.819, temperature) + (−0.022, mid-upper arm circumference) + (0.048 transformed oxygen saturation) + (1.793, parent concern) + (1.012, difficulty breathing) + (1.814, oedema) + (1.506, pallor (Table 2). The model achieved good predictive accuracy quantified by a cross validated AUROC curve of 86% (Figure 2A) and was well calibrated (Figure 2B).

**Table 2:** Summary of the triage model.

Variable	Standardized prompt	Estimate	*p*-value	Odds Ratio (95% CI)
(Intercept)		−32.888	<0.0001	
Transformed oxygen saturation*	Measure peripheral oxygen saturation using the mobile pulse oximeter (See SOP) (20).	0.048	<0.001	1.05 (1.02, 1.07)
Heart rate (bpm)	Record the patient's heart rate from previous pulse oximetry measurement (See SOP) (20).	0.016	<0.001	1.02 (1.01, 1.02)
Temperature (°C)	Measure and record axillary temperature (See SOP) (20).	0.819	<0.0001	2.27 (1.92, 2.69)
MUAC (mm)	Measure and record the child's mid-upper arm circumference (See SOP) (20).	−0.022	<0.0001	0.98 (0.97, 0.98)
Age (square root)	Enter age at admission in months.	0.252	<0.0001	1.29 (1.18, 1.40)
Parent concern	Ask: Do you think that your child needs admission because they are sicker than they have been in the past?	1.793	<0.0001	6.01 (3.79, 9.63)
Difficulty breathing	Is the child having difficulty breathing compared to a well child?	1.012	<0.0001	2.75 (1.97, 3.84)
Oedema	Does the child have pitting oedema on their feet, knees, or face?	1.814	<0.0001	6.14 (2.44, 10.76)
Pallor	Is the child pale at their palms, oral mucosa, or conjunctiva compared to their caretaker?	1.506	<0.0001	4.51 (2.69, 7.59)

* Transformed SpO_2_ computed using physiologically derived altitude adjusted virtual shunt formula: 70.103 × log 10(101.687 − spo2) − 55.833 (24). MUAC, mid-upper arm circumference.

### Risk stratification

The model achieved 91% (95% CI 88%, 94%) sensitivity at an 8% low-risk threshold and 92% (95% CI 90%, 94%) specificity at a 40% high-risk threshold (Figure 2A) (Table 3). The non-urgent and priority strata each comprised of approximately 40% of participants, while the remaining 20% were stratified as emergency (Table 3). The model achieved similar AUROC performance characteristics when applied to children under five years of age and when admission of any duration was used as the modelling outcome (Supplementary Figures S4). This demonstrates robustness across the range of ages and to adjustments of the admission outcome.

**Table 3:** Summary of risk stratification into three triage categories.

	**Non-urgent**	**Priority**	**Emergency**
**Risk threshold**	**≦0.08**	**>0.08 ≦0.40**	**>0.40**
**Participant and Outcome Stratification**			
Participants, *N* (%)	666 (41.3)	628 (39.0)	318 (19.7)
Admitted participants, *N* (%)	36 (5.4)	116 (18.5)	223 (70.1)
Length of stay < 24 h, *N* (%)	3 (0.45)	7 (1.1)	8 (2.5)
Length of stay 24–48 h, *N* (%)	7 (1.1)	32 (5.1)	70 (22.0)
Length of stay >48 h, *N* (%)	22 (3.3)	73 (11.6)	139 (43.7)
Readmitted participants*, N* (%)	7 (1.1)	13 (2.1)	18 (5.7)
* Within 48 h, N* (%)	5 (0.75)	5 (0.80)	13 (4.1)
Mortality, *N* (%)	0 (0.0)	2 (0.32)	9 (2.8)
Positive admission outcome, *N* (%)	34 (5.1)	110 (17.5)	220 (69.2)
**Performance Assessment** *			
True positive to false positive ratio	330:616	305:322	220:98
Sensitivity (95% CI)	0.91 (0.88, 0.94)	0.84 (0.80, 0.87)	0.59 (0.54, 0.64)
Specificity (95% CI)	0.50 (0.45, 0.61)	0.74 (0.72, 0.77)	0.92 (0.90, 0.94)
Negative predictive value (95% CI)	0.95 (0.94, 0.96)	0.94 (0.93, 0.95)	0.89 (0.86, 0.90)
Positive predictive value (95% CI)	0.35 (0.34, 0.38)	0.49 (0.46, 0.51)	0.69 (0.64, 0.73)
Negative likelihood ratio (95% CI)	0.18 (0.13, 0.24)	0.22 (0.17, 0.27)	0.43 (0.38, 0.49)
Positive likelihood ratio (95% CI)	1.80 (1.69, 1.92)	3.23 (2.91, 3.59)	7.50 (6.09, 9.23)

* Values computed using the upper limit, median, and lower limit of the risk threshold range for the non-urgent, priority, and emergency categories respectively. Results are pooled estimates of 2,000 bootstrap replicates. CI, confidence interval.

## Discussion

### Summary

The logistic triage model comprised of five objectively measurable (age, heart rate, temperature, oxygen saturation, MUAC) and four easily assessable (difficulty breathing, oedema, pallor, parent concern) predictor variables was developed using hospital admission as an indicator of acuity. Most predictors retained in this model are consistent with previous pediatric triage models (29, 30); and international guidelines (8). Parent concern is not currently included in ETAT + or used as a formal criterion to determine admission status. Nevertheless, there is growing evidence to support the role of parent concern in early recognition of critical illness in children (31). This model, when used with good clinical judgement and adequate quality control can help and guide clinical decision making in terms of patient prioritization. This model represents the first iteration of a triage tool that will be continuously updated to reflect the latest available data and adapted based on illness profiles and population dynamics.

The WHO has acknowledged that digital health interventions and systems that use patient data to drive health practices can lead to improved quality of care (32). An ETAT + mobile health intervention and an electronic IMCI algorithm implemented at primary health centres in Malawi and Burkina Faso, respectively, improved both triage and resource allocation (33, 34). Yet data-driven triage algorithms provide unique benefits over digitally implemented clinical guidelines. They enable quality improvement, are easily updated, and can be adjusted to meet setting-specific needs. Implementation of a standardized platform for data collection (21), and detailed standard operating protocols including a robust mechanism to capture follow up of discharged participants (20) will enable reproducibility required for model validation that is lacking in the current literature.

It should be noted that this triage model cannot and does not function to replace ETAT + but rather to serve as an adjunct to the patient prioritization process. ETAT + has been developed as a package of guidelines, training, and quality improvement in East Africa, and has become the national framework for facility-based pediatric care in this country. This triage algorithm helps guide patient prioritization at first contact for children who would otherwise be waiting in line for hours to be formally triaged and treated by health workers.

### Clinical interpretation

More than 90% of admitted participants were diagnosed with sepsis or infectious diseases thus validating the use of sepsis predictors as candidate predictors of critical illness in model development (Table 1). The high negative predictive performance of the model at the low-risk threshold supports its ability to exclude low-risk patients while the negative likelihood ratio indicates a 5.5-fold decrease in the odds of needing hospital admission for a patient classified as non-urgent (Table 3). A positive predictive value of 35% (95% CI 34%, 38%) was achieved at the high-risk threshold which, given the relatively low prevalence of positive admission outcomes, demonstrates capability of the model to correctly classify emergency patients. The positive likelihood ratio indicates a 7.5-fold increase in the odds of needing hospital admission for a patient classified as emergency (Table 3). Furthermore, 91.5% of participants admitted for at least 24 h, 78.3% of participants readmitted within 48 h, and 100% of deaths were contained in either the priority or emergency category (Table 3).

### Limitations

This model may not be generalizable to other contexts as it was developed from a very young cohort population, with high rates of malaria and malnutrition. While this type of cohort is typical of pediatric populations in low-income countries, model performance may be affected by varying age distributions and in settings without high rates of malaria and malnutrition.

A major limitation of the study was the use of hospital admission as a surrogate for severity of illness. The study cohort consisted only of children presenting with acute illnesses. To exclude admitted cases lacking severe illness, a duration of stay of at least 24 h was required to count as a positive outcome. A 7-day follow up call was conducted to capture children inadvisably sent home. Children who returned to a health facility and were readmitted within 48 h were also counted as positive outcomes. Mortality was also ascertained from the follow up call. Deaths in this 7-day period were minimal for both admitted (*n* = 9) and non-admitted (*n* = 2) participants and meaningful analysis could not be conducted. Both non-admitted cases that died at home were classified as emergency by the triage model and perhaps should have been considered positive outcomes. This however was unlikely to have impacted the results due to the low mortality rate of children sent home (0.6%).

Another limitation is the lack of external validation, which is needed to assess the model's clinical utility. The model is currently being evaluated in a multisite study that will include clinical implementation in low-resource hospitals across sub-Saharan Africa to assess performance in varied geographical locations, seasons, and with different disease prevalence and severity (16).

Finally, there are concerns regarding implementation of digital health systems in LMICs. These include limited availability of smartphones, computer infrastructure, and a continuous supply of electricity, in addition to staffing attitude and training challenges. In response, to complement the digital triage interventional study, we are conducting a cost-effectiveness analysis to maximize the benefit of implementing such technology in low resource hospitals (35). The WHO views digital health as an accessible and affordable solution for global health delivery in LMICs and an important tool in achieving sustainable development goals (36). Safe and appropriate scale up has been prioritized within national and global digital health strategies (37–39).

## Data Availability

The data studied, data dictionary, and code are available from the Pediatric Sepsis CoLab Dataverse, subject to an application meeting the ethical and governance requirements of the CoLab. Requests to access these datasets should be directed to jessica.trawin@cw.bc.ca (40).
