# Supplementary material for: Smart triage: Development of a rapid pediatric triage algorithm for use in low-and-middle income countries
Source: Front Pediatr. 2022 Nov 22;10:976870. doi: 10.3389/fped.2022.976870 (PMC9723221; doi:10.3389/fped.2022.976870)
Supplement: Supplementary file 2 [file Table2.pdf]

Supplementary Table S2: Candidate predictor variables, definitions, and response values (N=63).

Variable	Standardized Prompt	Response Values
**Patient Information**		
Age	Enter age at admission in months.	Integer (months)
Sex	Enter biological sex.	Female / Male
Length of Illness	Ask: How many days has the child been sick in this illness?	Integer (days)
Urgent referral	Ask: Was the child referred to this hospital from another health facility in the last 24 hours?	No / Yes
Previous admissions	Ask: Has the child been admitted to a hospital or health center in the last 6 months?	No / Yes
Number of previous admissions	Ask: How many times was the child admitted to the hospital or health centre in the last 6 months?	Number
Time since last hospitalization	Ask: How long ago was the child last admitted to a hospital or health center?	< 7 days / 7-14 days /14-30 days / 1 month / 2 months / 3 months / 4 months / 5 months / 6 months
**Vital Signs**		
Oxygen saturation	Measure peripheral oxygen saturation using the mobile pulse oximeter (See SOP).	Continuous (%)
Heart Rate	Record the patient's heart rate from previous pulse oximetry measurement (See SOP).	Continuous (bpm)
Respiratory Rate	Measure respiratory rate using the app provided (See SOP).	Continuous (breaths per minute)
Temperature	Measure and record axillary temperature (See SOP).	Continuous (degrees Celsius)
**Anthropometric Measurements**		
Weight	Measure and record the child's weight. (See SOP)	Continuous (kg)
Height	Measure and record the child's height. Measure length instead if younger than 2 years old (See SOP).	Continuous (cm)
Middle upper arm circumference	Measure and record the child's mid-upper arm circumference (See SOP).	Continuous (mm)
**Clinical Signs and Symptoms**		
	**Respiratory**	
Difficulty breathing	Is the child having difficulty breathing compared to a well child?	No / Yes
Stridor	Does the child have stridor? (High pitched sound from upper airway during inspiration)	No / Yes
Indrawing	Does the child have chest indrawing on inspiration? ("Drawing in" of lower chest during inspiration)	No / Yes

Flaring	Does the child have nasal flaring during inspiration? (Widening of nostrils during inspiration)	No / Yes
Grunting	Does the child have a grunt on expiration? (Short noise at each expiration)	No / Yes
Tracheal tug	Does the child have a tracheal tug on inspiration? (Downward movement of trachea during inspiration)	No / Yes
Accessory muscle use	Does the child use accessory muscles when breathing? (e.g., neck, chest, abdominal, or upper back muscles)	No / Yes
Wheezing	Does the child have a wheeze during expiration when listening with a stethoscope? (High pitched sound in chest during expiration)	No / Yes
Cough	Record if you have observed coughing from the child.	No / Yes
Central cyanosis	Are the child's lips or tongue a blue or dark blue color?	No / Yes
**Circulation**		
Supplemental oxygen	Is the child receiving supplemental oxygen?	No / Yes
Capillary refill time > 3 seconds (upper limb)	Apply pressure to a thumb or finger for 3 seconds to blanch it. Does it take more than 3 seconds to return to original pink color after you let go? (See SOP)	No / Yes
Cool peripheries	Are the child's hands cold?	No / Yes
Weak or absent radial pulse	Does the child's radial pulse feel weak or absent?	No / Yes
Pallor	Is the child pale at their palms, oral mucosa, or conjunctiva compared to their caretaker?	No / Yes
**Dehydration**		
Skin pinch	Pinch the child's skin in the lower arm until it is tented, then release it. Does it take longer than 2 seconds to return to baseline?	No / Yes
Sunken eyes	Does the child's eyes appear to sink into their sockets?	No / Yes
No tears while crying	Ask: Has the child stopped making tears when he/she is crying?"	No / Yes
Dry oral mucosa	Does the child's oral mucosa (not lips) appear dried out?	No / Yes
**Gastrointestinal/Genitourinary**		
Vomiting	Ask: Does the child throw up everything they eat or drink?	No / Yes
Diarrhoea	Ask: Has the child had diarrhoea 3 or times per day since getting sick?	No / Yes
Chronic diarrhea	Ask: Did the child have diarrhea for more than two weeks?	No / Yes
Dysentery	Ask: Has the child had blood in their stools?	No / Yes
**Neurologic**		

AVPU	Assess and select consciousness level using the AVPU scale (See SOP).	Alert / Responds to voice / Responds to pain / Unresponsive
Convulsions (reported, history of)	Ask: Has the child had convulsions before? (See SOP).	No / Yes
Convulsions (actively, now)	Was the child actively convulsing during this assessment?	No / Yes
Irritable	Has the child been crying uncontrollably throughout the interview, even before you approached them?	No / Yes
Inability to drink or breastfeed	Ask: Has the child been too sleepy or tired to breastfeed, drink, or eat for more than 6 hours?	No / Yes
**Malnutrition**		
Oedema	Does the child have pitting oedema on their feet, knees, or face?	No / Yes
Severe wasting	Does the child seem wasted?	No / Yes
**Infection**		
History of fever	Ask: Does the child have a history of fever?	No / Yes
Rash	Does the child have a rash?	No / Yes
Infective lesion	Does the child have an infective lesion on the skin, eye or ear?	No / Yes
**Trauma**		
Major trauma	Has the child suffered trauma needing a designated trauma team or urgent surgery? OR Does he/she have a penetrating injury, pelvic or long bone fracture, or head or neck injury?	No / Yes
Burns	Has the child suffered a burn resulting in skin breakdown?	No / Yes
Poisoning	Has the child ingested a chemical or drug not intended for ingestion by the child?	No / Yes
Severe pain	Does the child seem to be in severe pain?	No / Yes
**Other**		
Parent concern	Ask: Do you think that this child may need admission because he is sicker than he has been in the past?	No / Yes
**Sociodemographic Information**		
Child HIV status	Record HIV status if records available. Otherwise ask: Has your child tested positive for HIV?	No / Yes
Maternal HIV status	Record HIV status if records available. Otherwise ask: Has the child's mother tested positive for HIV?	No / Yes
Primary caregiver	Ask: Who is the primary caregiver of the child?	Mother / Father / Grandparent / Other relative / Non-relative / Daycare

Mother alive	Ask: Is the child's mother still alive?	No / Yes
Maternal education	Ask: What is the highest level of school reached by the child's mother? Read out options.	No school / Primary / Secondary / Post-secondary / Don't know
Maternal age	Ask: How old is the child's mother?	Integer (years)
Boil all drinking water	Ask: Do you always boil water before the child drinks it?	No / Yes
Water source	Ask: Where does the child get most of his/her drinking water from? Read out options.	Bottled / Tap / Borehole / Protected spring / Open source / Slow running water / Fast running water / Don't know
**Laboratory Testing**		
HIV	Enter HIV test result.	Negative / Positive / Not done
Malaria	Enter Malaria test result.	Negative / Positive / Not done
