# Supplementary material for: Smart triage: Development of a rapid pediatric triage algorithm for use in low-and-middle income countries
Source: Front Pediatr. 2022 Nov 22;10:976870. doi: 10.3389/fped.2022.976870 (PMC9723221; doi:10.3389/fped.2022.976870)
Supplement: Supplementary file 3 [file Table3.pdf]

Supplementary Table S3: Summary of predictor variables stratified across participants with and without a positive admission outcome.

Factor variables	Missing	Events among participants with an admission outcome (N)	Events among participants with no admission outcome (N)	Total events (N)	Event admission rate (%)	Odds ratio (95% CI)	p-value
**Patient Information**							
Male sex	3	204	625	829	0.246	1.26 (1.00, 1.59)	0.055
Previous admissions	4	98	194	292	0.336	2.00 (1.51, 2.64)	<0.0001
Urgent referral	5	60	37	97	0.619	6.46 (4.23, 9.99)	<0.0001
**Clinical Sings and Symptoms**							
**Respiratory**							
Difficulty breathing	4	154	206	360	0.428	3.71 (2.87, 4.79)	<0.0001
Stridor	5	6	11	17	0.353	1.45 (0.59, 3.84)	0.469
Indrawing	5	48	36	84	0.571	4.00 (2.33, 6.35)	<0.0001
Flaring	5	40	24	64	0.625	4.93 (2.94, 8.44)	<0.0001
Grunting	5	13	8	21	0.619	4.44 (1.85, 11.32)	0.001
Tracheal tug	5	12	10	22	0.545	3.26 (1.39, 7.80)	0.006
Accessory muscle use	5	44	26	70	0.629	5.07 (3.08, 8.50)	<0.0001
Wheezing	5	25	36	61	0.410	1.91 (1.11, 3.23)	0.0165
Cough	5	130	313	443	0.293	1.17 (0.89, 1.53)	0.251
Central cyanosis	4	5	2	7	0.714	NA	<0.0001
**Circulation**							
Supplemental oxygen	3	9	1	10	0.900	NA	0.001
Capillary refill time > 3 seconds (upper limb)	4	50	18	68	0.735	10.88 (6.38, 19.40)	<0.0001
Cool peripheries	0	39	10	49	0.796	2.84 (0.89, 9.05)	0.074
Weak or absent radial pulse	4	0	1	1	0.000	NA	0.972
Pallor	8	83	39	122	0.680	9.15 (6.17, 13.81)	<0.0001

**Dehydration**							
Skin pinch	4	18	191	209	0.086	1.98 (0.98, 3.88)	0.049
Sunken eyes	4	38	44	82	0.463	2.84 (1.80, 4.46)	<0.0001
No tears while crying	4	11	20	31	0.355	1.70 (0.78, 3.53)	0.162
Dry oral mucosa	4	29	26	55	0.527	0.31 (0.18, 0.49)	<0.0001
**Gastrointestinal/Genitourinary**							
Vomiting	4	103	159	262	0.393	2.70 (2.03, 3.58)	<0.0001
Diarrhoea	4	90	248	338	0.266	1.32 (1.00, 1.74)	0.0458
Chronic diarrhoea	4	10	20	30	0.333	1.43 (0.62, 3.11)	0.386
Dysentery	4	10	21	31	0.323	1.35 (0.59, 2.93)	0.458
**Neurologic**							
**AVPU:**	4						
1. alert		355	1242	1597	0.222	0.17 (0.05, 0.54)	0.003
2. responds to voice		3	0	3	1.000	NA	NA
3. responds to pain		5	3	8	0.625	NA	NA
4. unresponsive		0	0	0	0.000	NA	NA
Convulsions (reported, history of)	4	5	1	6	0.833	NA	0.155
Convulsions during assessment, (observed)	4	1	2	3	0.333	NA	0.273
Irritable	8	42	73	115	0.365	2.09 (1.40, 3.11)	<0.0001
Inability to drink or breastfeed	9	97	91	188	0.516	4.62 (3.37, 6.34)	<0.0001
**Malnutrition**							
Oedema	8	19	11	30	0.633	6.19 (2.97, 10.58)	<0.0001
Severe wasting	0	19	23	42	0.452	2.82 (1.21, 6.27)	0.0126
**Infection**							
History of fever	8	321	899	1220	0.260	3.08 (2.19, 4.44)	<0.0001
Rash	8	50	273	323	0.150	0.56 (0.41, 0.78)	<0.001
Infective lesion	8	10	70	80	0.130	0.48 (0.23, 0.89)	0.0304
**Trauma**							

Major trauma	8	0	3	3	0.000	NA	NA
Burns	8	2	15	17	0.118	0.45 (0.07, 1.62)	0.296
Poisoning	8	4	7	11	0.364	1.96 (0.51, 6.56)	0.282
Severe pain	8	110	72	182	0.604	7.07 (5.12, 9.84)	<0.0001
**Other**							
Parent concern	4	93	42	135	0.69	9.85 (6.73, 14.64)	<0.0001
**Sociodemographic Information**							
Child HIV status	4	144	441	585	0.246	0.92 (0.23, 0.32)	0.142
Maternal HIV status	4	11	42	53	0.208	0.93 (0.45, 1.76)	0.828
**Primary caregiver:**	4						
1. mother		333	1186	1519	0.219	0.28 (0.25, 0.32)	0.012
2. father		9	10	19	0.474	2.19 (1.27, 8.04)	0.008
3. grandparent		19	31	50	0.380	0.67 (0.16, 2.03)	0.527
4. other relative		3	16	19	0.158	NA	NA
5. non-relative		0	1	1	0.000	NA	NA
Mother alive	4	5	5	10	0.500	0.29 (0.08, 1.04)	0.051
**Maternal education:**	4						
1. no school		16	44	60	0.267	1.15 (0.64, 2.15)	0.655
2. primary		151	362	513	0.294	0.75 (0.42, 1.40)	0.34
3. secondary		170	621	791	0.215	0.31 (0.15, 0.64)	0.001
4. post-Secondary		24	212	236	0.102	0.39 (0.07, 2.34)	0.524
Boil all drinking water	4	199	885	1084	0.184	0.49 (0.38, 0.62)	<0.0001
**Primary water source for drinking:**	4						
1. bottled		1	4	5	0.200	NA	NA
2. municipal/tap		223	1023	1246	0.179	1.13 (0.59, 7.70)	0.900
3. bore hole		103	143	246	0.419	0.29 (0.06, 2.02)	0.347
4. protected spring		30	62	92	0.326	0.44 (0.02, 3.14)	0.562
5. open source		3	3	6	0.500	NA	NA
6. slow running water		1	2	3	0.333	NA	NA

7. fast running water		1	0	1	1.000	NA	NA
**Laboratory Testing**							
HIV		2	1	3	0.667	NA	
Malaria		166	128	294	0.565	0.99 (0.99, 0.99)	<0.0001
**Continuous Variables**	**Missing**	**Median (IQR) for participants with an admission outcome**	**Median (IQR) for participants without an admission outcome**	**Odds Ratio (95% CI)**			**p-value**
**Patient Information**							
Age (months)	4	16.5 (32.9)	13.1 (21.8)	1.01 (1.00, 1.01)			<0.01
Length of illness (days)	0	2.5 (2.0)	3.0 (2.0)	1.00 (0.98, 1.01)			0.931
**Vital Signs**							
Oxygen saturation	10	98.1 (2.9)	99.0 (2.1)	0.86 (0.83, 0.90)			<0.0001
Transformed oxygen saturation*	10	0 (0)	0 (0)	1.06 (1.04, 1.09)			<0.0001
Heart Rate	10	149.5 (31.2)	138.0 (27.0)	1.03 (1.02, 1.03)			<0.0001
Respiratory Rate	6	50 (22.0)	43.0 (18.0)	1.03 (1.02, 1.04)			<0.0001
Temperature	4	37.8 (1.9)	36.8 (0.7)	2.64 (2.32, 3.02)			<0.0001
**Anthropometric Information**							
Height (cm)	9	77 (22.5)	74.0 (21.1)	1.01 (1.00, 1.01)			<0.01
Weight (kg)	4	9.9 (4.2)	9.2 (4.6)	1.01 (0.99, 1.03)			0.47
MUAC (mm)	3	140 (24.0)	143.0 (23.0)	0.99 (0.98, 1.00)			<0.001
**Sociodemographic Information**							
Maternal Age	2	26 (7.0)	26 (7.0)	1.00 (0.98, 1.02)			0.903

**Transformed Spo2 computed using physiologically derived altitude adjusted virtual shunt formula: 70.103*log10(101.687-spo2)-55.833.*
