# Supplementary material for: Smart triage: Development of a rapid pediatric triage algorithm for use in low-and-middle income countries
Source: Front Pediatr. 2022 Nov 22;10:976870. doi: 10.3389/fped.2022.976870 (PMC9723221; doi:10.3389/fped.2022.976870)
Supplement: Supplementary file 4 [file Datasheet1.pdf]

Supplementary Figures S4: Model performance in selected sub-cohorts.

Figure 1: Cross validated receiver operating characteristic curve of the triage model in the (a) under 5 cohort and (b) the entire cohort.

(a) **(b)**

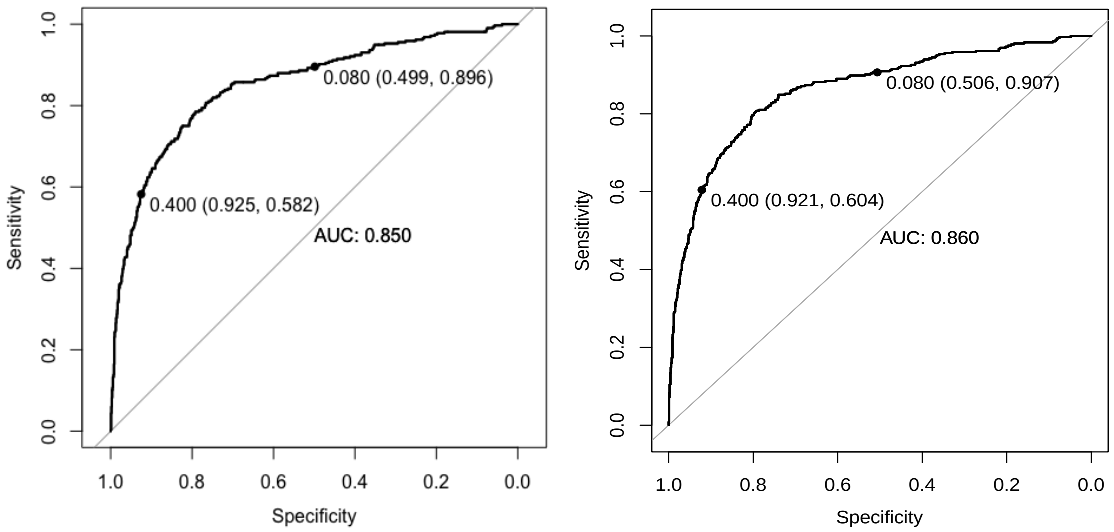

Points represent low risk (8%) and high risk (40%) thresholds. AUC, area under the curve.

Figure 2: Calibration plot of the triage model for (a) the under 5 cohort and (b) the entire cohort.

(a) **(b)**

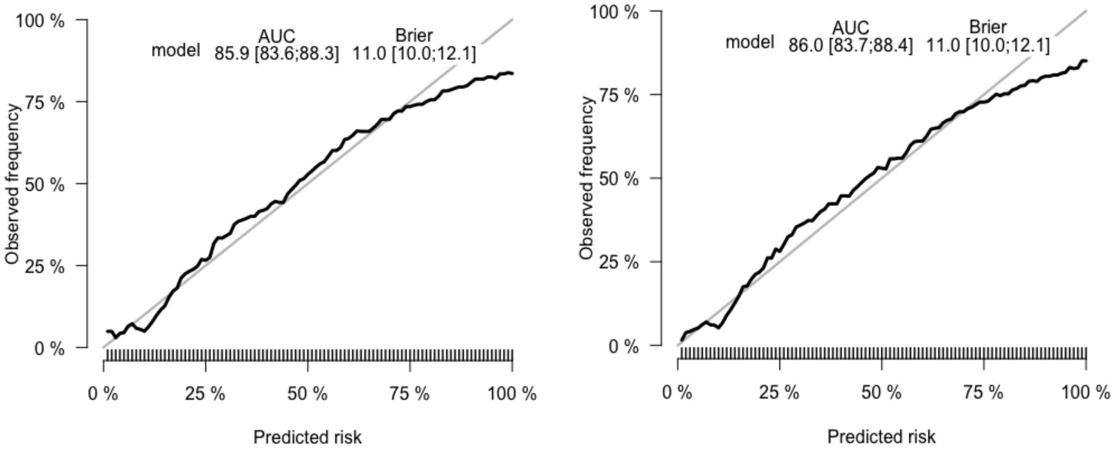

The 45-degree straight line corresponds to the line of perfect calibration on which model predicted risks coincide with the observed frequency. AUC, area under the curve (%); Brier, Brier score (%).

Figure 3: Cross validated receiver operating characteristic curve of the triage model with outcomes defined as (a) hospital admission (b) hospital admission > 24 hours OR readmission within 48 hours.

(a) **(b)**

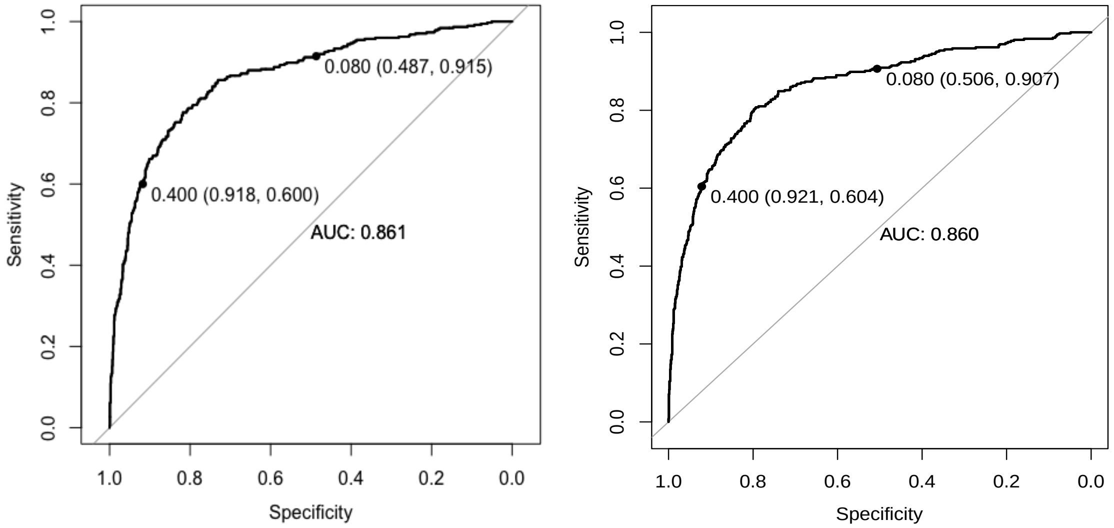

Points represent low risk (8%) and high risk (40%) thresholds. AUC, area under the curve.

Figure 4: Calibration plots of the triage model with outcomes defined as (a) hospital admission (b) hospital admission > 24 hours OR readmission within 48 hours.

(a) **(b)**

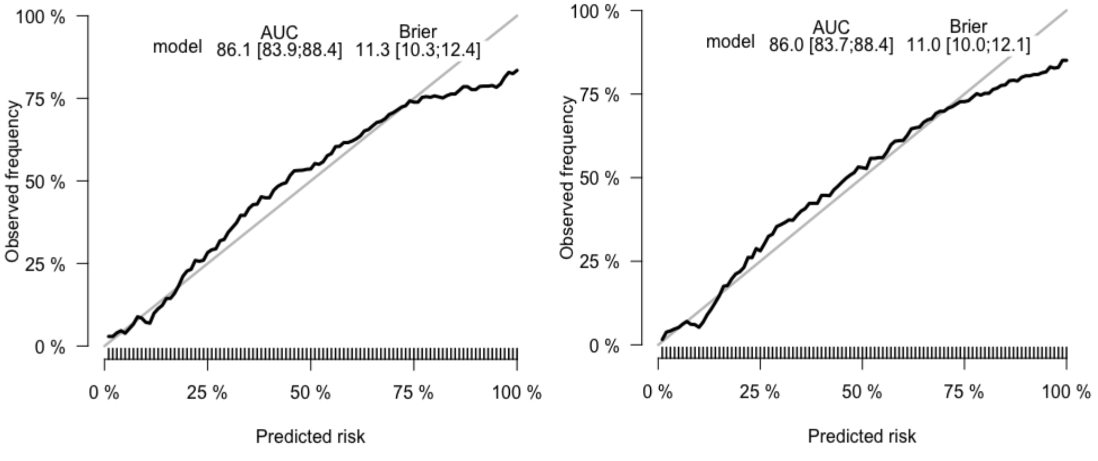

The 45-degree straight line corresponds to the line of perfect calibration on which model predicted risks coincide with the observed frequency. AUC, area under the curve (%); Brier, Brier score (%).
